# Predicting the Future Impact of Droughts on Ungulate Populations in Arid and Semi-Arid Environments

**DOI:** 10.1371/journal.pone.0051490

**Published:** 2012-12-17

**Authors:** Clare Duncan, Aliénor L. M. Chauvenet, Louise M. McRae, Nathalie Pettorelli

**Affiliations:** 1 Institute of Zoology, Zoological Society of London, London, United Kingdom; 2 Division of Biology, Imperial College London, Ascot, United Kingdom; University of Sydney, Australia

## Abstract

Droughts can have a severe impact on the dynamics of animal populations, particularly in semi-arid and arid environments where herbivore populations are strongly limited by resource availability. Increased drought intensity under projected climate change scenarios can be expected to reduce the viability of such populations, yet this impact has seldom been quantified. In this study, we aim to fill this gap and assess how the predicted worsening of droughts over the 21^st^ century is likely to impact the population dynamics of twelve ungulate species occurring in arid and semi-arid habitats. Our results provide support to the hypotheses that more sedentary, grazing and mixed feeding species will be put at high risk from future increases in drought intensity, suggesting that management intervention under these conditions should be targeted towards species possessing these traits. Predictive population models for all sedentary, grazing or mixed feeding species in our study show that their probability of extinction dramatically increases under future emissions scenarios, and that this extinction risk is greater for smaller populations than larger ones. Our study highlights the importance of quantifying the current and future impacts of increasing extreme natural events on populations and species in order to improve our ability to mitigate predicted biodiversity loss under climate change.

## Introduction

In light of the current global extinction crisis, understanding how and where drivers of population decline will take effect has never been more important [1]. Climate change is expected to be a major driver of species extinctions in the 21^st^ century [2], [3]. Average changes in greenhouse gas concentrations are expected to produce directional changes in climatic conditions, and increase the level of inter-annual variability in these conditions [4]. Droughts are a significant component of such climatic variability, and can have a devastating impact on animal populations [5]–[8]. Through processes such as recurrent reductions in population numbers and the consequent genetic effects caused by demographic bottlenecks [5], droughts have the potential to lead populations, and entire species, to extinction.

In a recent publication, the Intergovernmental Panel on Climate Change (IPCC) reported a likely increase in droughts over the 21^st^ century in various regions of the world, including southern Europe and the Mediterranean, central Europe, central North America, Central America and Mexico, northeast Brazil, and southern Africa [9]. This predicted increase is a potential cause for conservation concern: although the impact of droughts on population size fluctuations has been assessed by many [10]–[13], the potential impact of expected changes in drought conditions on wildlife populations has almost never been quantified (but see [14]). Moreover, to date no comparative study has been conducted to assess the impact of future changes in drought conditions for species exhibiting contrasting life histories. The risk of extinction of species in response to various threats is partially shaped by their intrinsic biological characteristics, e.g., body mass, feeding strategy, reproductive strategy, territoriality and home range size [15], [16]. Certain life history traits can be expected to make species more susceptible to increased droughts, such as strong dependence on permanent water-sources [17], [18]; obligate grazing or mixed feeding (due to whole or partial dependence on drought-intolerant food species [19], [20]); sedentary behaviour (due to being unable to escape the effects of drought conditions on resource availability [20]–[21]). However, how possessing these traits will shape the future susceptibility of populations to changes in climatic conditions is currently unknown. Such information is yet deemed necessary to improve our ability to mitigate predicted biodiversity loss under climate change.

As highlighted by the IPCC 2012 report [9], the need to quantitatively assess how predicted changes in drought conditions are likely to impact wildlife is particularly great for populations inhabiting semi-arid and arid regions. Primary productivity in these environments is already heavily limited by precipitation [22]. As a result, even a slight increase in drought duration, intensity or frequency in these regions has the potential to severely impact resource availability and thus herbivore abundance [12], [23]–[25]. Increased drought conditions may also indirectly impact herbivore populations in these habitats as reduced forage and water availability can lead to increased vulnerability to predation, due to e.g., increased densities at sparse water points [26]–[27]. This study thus proposes to quantify the impact of future changes in drought conditions on future growth rate of terrestrial ungulate species with contrasted life histories inhabiting arid and semi-arid environments. Based on the knowledge available on ungulate susceptibility to drought [18]–[23], we hypothesised that population growth rates of species that are grazers or mixed feeders (H1), and that are relatively sedentary (H2) should show a significant negative relationship with drought intensity, and be more negatively impacted by drought intensity than population growth rates of other species groups.

## Materials and Methods

### Drought Data

An extreme natural event, such as a drought, can be defined as an event that is rare within its statistical reference distribution. It is normally as rare or rarer than the 10^th^ or 90^th^ percentile [4], [28], and can be quantified in relation to a specific (possibly impact-related) threshold [9]. However, it is difficult to quantitatively define a drought as there are several different forms (e.g., meteorological drought, agricultural drought and hydrological drought; see [29]). As a result, several different drought indices have been developed [30], [31]. One index developed to quantify meteorological drought is the Palmer Drought Severity Index (PDSI; [32]). The PDSI is a common meteorological drought index [33], which has been used to quantify both historical and projected long-term trends in global aridity (see [34]). The PDSI is a standardized index, incorporating both previous and current moisture supply (precipitation) and demand (potential evapotranspiration, PE), which ranges from −10 (dry) to +10 (wet).

Palmer’s original PDSI was calibrated using fixed coefficients from limited data from the central United States, and to improve spatial comparability several attempts have been made to recalibrate the PDSI index since (see [35]). Self-calibrating PDSI (sc_PDSI) [36] has been found to be more spatially comparable than Palmer’s original PDSI, while calculation of PE using the more sophisticated Penman-Monteith equation (PDSI_pm) as opposed to the original Thornwaite equation, has also improved the efficiency of the PDSI [35]. We used global PDSI data (hereafter sc_PDSI_pm) for the years 1970 to 2005. It is calculated using observed or modelled monthly air surface temperature and precipitation, calibrated with Penman-Monteith PE based on historical data and gridded to a 2.5°x2.5° grid ([35], [36];http://www.cgd.ucar.edu/cas/catalog/climind/pdsi.html).

### Study Species and Population Data

Only ungulate species occurring in arid and semi-arid areas where droughts are predicted to become more common and more intense over the 21^st^ century were considered for this analysis. To identify relevant species, we first compiled ungulate species distribution data (genera Artiodactyla, Perissodactyla, Proboscidea) from the International Union for the Conservation of Nature (IUCN) mammal species dataset (Geographic Information Systems data available at http://www.iucnredlist.org/technical-documents/spatial-data), and then overlaid these species distribution maps with a map of global arid areas of the Köppen-Geiger climate classification ([37]; Geographic Information Systems data available at http://koeppen-geiger.vu-wien.ac.at/shifts.htm).

Time-series abundance data for 1970–2005 for the ungulate populations found in arid and semi-arid areas were collated from the WWF/ZSL Living Planet Index (LPI) database [38]. The database is comprised of yearly abundance data (either population size estimates, population density, relative abundance, biomass, index data, proxy data, samples or measures per unit effort) for vertebrate populations, collated from data in published scientific literature, unpublished reports and online databases. Data is only included in the database if the method of collection or estimation, the geographic location of the population, and the units of measurement are known, and if the data source is referenced and traceable.

As our study focused on the impact of drought on population growth rate, abundance records were only included in the analysis when population estimates were for two consecutive years; i.e. if two abundance records had a one or more years gap between them they were not included. Only species for which the sample size of growth rates over all populations was greater than 20 observations were included in the analysis. Moreover, populations for which the initial starting abundance record was smaller than the average herd size range for that given species were removed in order to ensure that we did not include unstable populations in our analysis. In addition, individual populations for which heavy management practices (e.g. African elephant (*Loxodonta africana*), African buffalo (*Syncerus caffer*), blue wildebeest (*Connochaetes taurinus*) and impala (*Aepyceros melampus*) in Kruger National Park, South Africa), or poaching activities (e.g. African elephant in Ruaha National Park, Tanzania and white rhinoceros (*Ceratotherium simum*) in Garamba National Park, Democratic Republic of Congo) were known to occur over the years of the abundance record (i.e. where references in the literature confirm the presence of such processes) were also excluded in order to eliminate potentially biased abundance data. Then, populations for which estimates were collated from a number of different sources and using a number of different estimation methods were also omitted due to the resulting high level of sampling bias. Finally, populations that contained growth rates higher than that which is physiologically possible for the given species (e.g., higher than possible if all members of the population in a given year were female, each gave birth to the maximum number of offspring possible for that species in one year, and there was no mortality) were not used (n = 4). Such high growth rates are very likely attributable to population increases caused by alternative processes such as immigration, the intentional introduction of individuals into national parks, or incorrect estimates of population size.

The resulting dataset comprised time-series abundance data from 71 populations of 12 ungulate species (Table S1). Most ungulate species currently covered by the LPI database occur in eastern and southern Africa, due to increased sampling effort in these areas. As a result, most of our study species and populations also occur in these regions. The geographic coordinates for each population were taken from the LPI database [38], and monthly sc_PDSI_pm values for each location were collated from the 2.5°x2.5° grid pixel in which the population fell and the years in which they were surveyed.

### Calculating Drought Indices

A drought can be described by three axes: duration, frequency, and intensity [39]. Drought duration refers to the timescale of the drought occurrence, e.g., the length of the drought episode. Frequency refers to the average interval (or distance) between drought events at a given location, which can vary between two years in extreme arid regions and 100 years in extremely wet regions [39]. Intensity refers to the extent of the precipitation deficit, and is usually calculated in relation to the duration as the cumulative moisture deficiency across the drought duration [39], [40].

For this study, a year is classified as a “drought year” if the sc_PDSI_pm value of at least one month within that year is below the value of the 10th percentile of its statistical reference distribution at a particular location (e.g., threshold *θ*). Drought intensity then refers to the number of consecutive months within a given “drought year” in which *q*<*θ* (where *q* is the sc_PDSI_pm value for a given month). In addition, drought frequency (or recurrence interval) is defined as the average distance (in years) between “drought years” across all study locations for each species between 1970 and 2005.

In order to test the impact of changes in drought conditions on the population growth rate of ungulate species in arid and semi-arid areas, we developed four potential predictor variables of drought: the total number of months of the preceding year (*T* ), and the preceding two years (*T_t_*_2_), in which *q*<*θ*; the maximum number of consecutive months of the preceding year (*C* ), and the preceding two years (*C_t_*_2_), in which *q*<*θ*. The variables *C* and *C_t_*_2_ were developed based on the definition of a drought event provided by Sheffield and Wood [40]. The variables *T* and *T_t_*_2_ were developed in order to create indices of drought that incorporated potential small breaks in drought occurrence, which could not be incorporated under the variables *C* and *C_t_*_2_. The correlation between our chosen drought index and both annual average PDSI and annual modal PDSI across all study populations was tested using the Spearman’s rank correlation. All analyses were carried out in R v. 2.14.2 [41].

### Modelling the Future Impact of Drought on Ungulate Populations

#### Establishing a link between drought indices and growth rates

For all ungulate species considered, we calculated observed population growth rates (*r_t_*) for a given year *t* between 1970 and 2005 as the change in abundance over time, normalised by the natural logarithm:
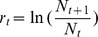
where *N_t_* is the population size at time *t*, and *N_t_*_+1_ is the population size at time *t*+1.

We then carried out single predictor regressions of observed population growth rate (*r_t_*) against *C*, *T*, *C_t_*_2_ and *T_t_*_2_ using linear mixed effect models with population location and species as random effects in order to identify the best predictor of *r_t_* for all the populations and species considered. This helped us identify the best drought indicator variable of the four.

In order to test our two hypotheses, we then created four subset species groups based on descriptions of individual species diet and movement behaviour in the literature [42]: (1) non-sedentary species (e.g., species that are described as being nomadic, migratory, displaying seasonal movements, or extremely wide-ranging) that wholly or partially depend on drought-intolerant food species (e.g., species that are described as pure grazers or ‘mostly’ grazers, and mixed feeders) (hereafter MG), (2) non-sedentary species that do not depend on drought-intolerant food species (e.g., species that are described as pure browsers or ‘mostly’ browsers, and omnivores) (hereafter MB), (3) sedentary species that wholly or partially depend on drought-intolerant food species (hereafter SG), and (4) sedentary species that do not depend on drought-intolerant food species (hereafter SB). We then carried out single predictor regressions of population growth rate (*r_t_*) for each of these groups against the best drought indicator variable; we used linear mixed models with population location and species as random effects.

#### Model description

For each group of species (MG, MB, SG, and SB) for which our best performing variable of drought intensity was found to be a significant predictor of *r_t_* we developed species-specific stochastic population models to predict the impact of future drought conditions on their viability. Because each species was divided into several populations, for which records of abundance were different, we built the model to project each population separately, e.g., the initial population size *N*_0_ was different per population. Our model took the form:
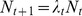
where



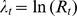
and



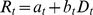



*R_t_* is the modelled population growth rate at time *t*. *b_t_* is a coefficient describing the impact of drought conditions (*D_t_*) on the modelled growth rate for each group (MG, MB, SG, and SB): it was estimated using the outputs of the linear mixed effects models for each group of ungulate species. *a_t_* is the average growth rate in the absence of drought: this coefficient was estimated using the observed average growth rate (*r_av_*) for each individual species. *D_t_* describes the drought conditions of a given year *t* and reflects the structure of the best drought indicator variable found above.

While modelling *D_t_* we aimed to reproduce observed drought patterns in our data. To do so, we first determined the average lengths of drought and non-drought episodes per years (i.e., the average number of months in droughts or not in drought per years) across all populations of the species for which we built a stochastic population model. This was to be able to simulate the length (in months) of a drought episode if a given modelled year was in drought. To determine if a modelled year experienced drought or not, we first generated an initial value *D_t_* at time *t*_0_ by comparing a random number sampled from a uniform distribution between 0 and 1, to an ‘initial threshold’ (Table S2). This initial threshold number was the probability that a given year *t* would be in drought based on observed drought patterns for each species between 1970 and 2005. If the random number was greater than the threshold number, year *t* was considered to be not in drought (*D_t_* = 0). If the random number was lower than the threshold number, year *t* was considered to be in drought, and *D_t_* was assigned a value between 1 and 12 to represent a number of months in drought. The value of *D_t_* when in drought was randomly sampled from the observed distribution of our best performing drought indicator variable for the given group of species.

**Figure 1:**
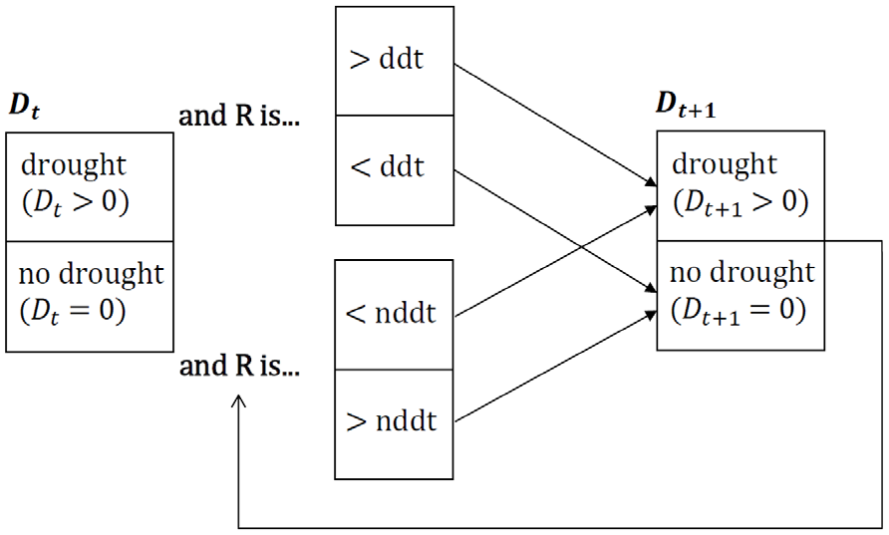
Model generation of *D_t_*_+1_ at each time step, where R is a random number sampled from a uniform distribution, and ‘ddt’ and ‘nddt’ correspond to the ‘drought distance threshold’ and the ‘non-drought distance threshold’ respectively.

Then, at each time-step (i.e., each simulated year) we assigned a drought or non-drought status to the year based on new ‘distance thresholds’, representative of observed drought event length (in years) and observed non-drought event length (in years). *D_t_*_+1_ was computed by comparing a random number (again from a uniform distribution between 0 and 1) to these ‘distance thresholds’; if in the preceding year, at time *t*, *D_t_* >0, then *D_t_*_+1_ would be computed by comparing the random number generated to the ‘drought distance threshold’ (Figure 1). The drought distance threshold for each group of species is the observed probability for each group that if year *t* was in drought, then year *t*+1 would also be in drought. However, if at time *t*, *D_t_* = 0, then *D_t_*_+1_ would be determined by comparing the random number generated to the ‘non-drought distance threshold’ (Figure 1). Similarly, the non-drought distance threshold is the observed probability for each group of species that if year *t* was not in drought (*D_t_* = 0), then year *t*+1 would also not be in drought. If *D_t_* = 0 and the random number generated was greater than the non-drought distance threshold, then *D_t_*_+1_ = 0, or if *D_t_* was >0 and the random number generated was greater than the drought distance threshold then the value of *D_t_*_+1_ was again generated from a random sample based on the probability distribution of our best performing drought variable, described above.

To test how well how the stochastic population models performed, we calculated the r-squared values between the observed abundances between 1970 and 2005 and the model-predicted abundances for each population of each species. Moreover, we assessed the ability of the models to reproduce observed patterns in the average length of drought and non-drought events (in years) by performing Wilcoxon signed-rank tests for the 95% confidence intervals of the median for both the observed and model predicted drought and non-drought event lengths (in years).

#### Simulations

For all groups of species for which a significant relationship was found between growth rates and the best performing drought indicator variable, we used emissions scenario predictions for the region in which each species occurs to train our species-specific model simulations. Three scenarios of future drought occurrence were considered: the first scenario assumed the continuation of current conditions (hereafter 20C). The two other scenarios were based on Sheffield and Wood’s projections [40] for the southern African region. Under the second scenario, a doubling of short-term (4–6 months) droughts detectable by 2025 was considered (hereafter B1); under the third scenario, a tripling of short-term droughts detectable by 2040 was considered (hereafter A2) [40]. Changes in occurrence of medium-term (7–11 months) droughts were not investigated by Sheffield and Wood [40]. As we were modelling drought occurrence on a yearly basis (e.g., 1–12 months length), we made the assumption that medium-term droughts would exhibit similar increases as short-term droughts under scenarios B1 and A2, which make our results a cautious underestimate of future drought occurrence.

For each population of each species, we ran a model for which the initial abundance corresponded to that population’s first abundance record. Because the model was stochastic, it was run for 5000 simulations in order to generate a large number of possible population trajectories from which to draw a mean observation. The total number of time-steps for each population was (1) 2005-*t*_0_ (as each population had a different starting year *t*_0_), in order to reproduce observed abundance, and (2) 2099-*t*_0_, in order to model future abundance up to 2099 under different climate change scenarios. A given population was considered extinct when its size was ≤5 individuals.

## Results

Three (*C*, *T* and *T_t_*_2_) of the four derived indices of drought conditions were found to show a significant negative relationship with observed growth rates (*r_t_*) across all study species (*C*: slope = −0.01, *p*<0.01; *T*: slope = −0.01, *p*<0.01; *T_t_*_2_: slope = −0.01, *p* = 0.03), while the relationship between the maximum number of consecutive months of drought (*q*<θ) over the preceding year (*C_t_*_2_) and observed growth rates (*r_t_*) was not significant (slope = −0.01, *p* = 0.11). All four drought indices were highly correlated with each other, with *C_t_*_2_ showing the lowest correlations with all other variables (Table S3), potentially explaining the non-significance of its relationship with *r_t_*. The two measures of drought intensity over the preceding year (*C* and *T*) displayed the most significant relationships with observed growth rates (*r_t_*). Thus, based on previous definitions of drought occurrence in the literature [40], we elected to use *C* only in further analyses. There was a high degree of correlation between *C* and the annual average PDSI (rho = −0.60, *p*<0.001), and *C* and the annual modal PDSI (rho = −0.59, p<0.001) across all study populations.

**Figure 2:**
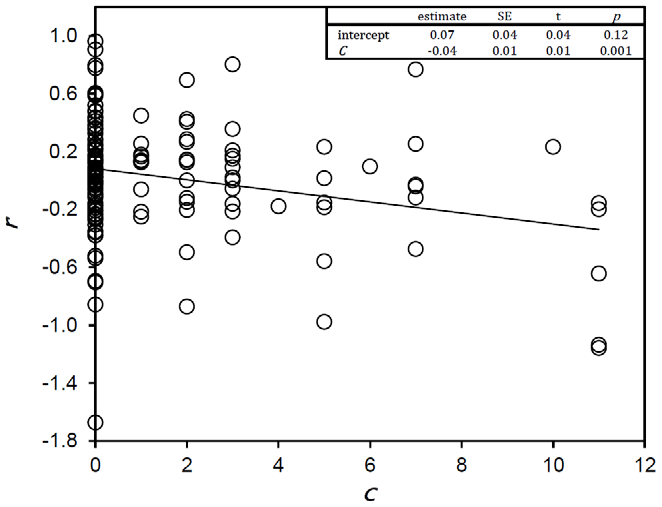
Number of consecutive months of the preceding year in which *q<* *θ* (***C***) as a predictor of growth rates (*r*) for all sedentary, grazing or mixed feeding species (4 species, n = 148).

As expected (H1 & H2), species with different life histories did not exhibit the same level of susceptibility to drought conditions: when modelling observed growth rates as a function of *C* for each species group (SB, SG, MB, MG), the only group of species for which *C* showed a significant negative relationship with growth rates was the group of sedentary species that either wholly or partially depend on drought-intolerant food species (SG group; slope = −0.04, *p* = 0.001) (Figure 2; Figure S1). Species that fell within this group were buffalo (*Syncerus caffer*), impala (*Aepyceros melampus*), hartebeest (*Alcelaphus buselaphus*) and waterbuck (*Kobus ellipsiprymnus*), and the population dynamics impacts of future increases in drought conditions were therefore only investigated for these four species.

The stochastic population models showed a certain level of heterogeneity in their ability to mimic observed abundances across individual species and across individual populations. For example, our model explained 70% of the variance in abundance of buffalo in the Serengeti-Mara ecosystem and in Addo Elephant Park, but only 20% in the Narok District; it explained 36% of variance in abundance of waterbuck in Malilangwe Wildlife Reserve but only 2% in Kruger National Park (Table 1). However, observed average drought (observed: pseudomedian = 2.0, lower 95% Confidence Interval = 1.5, upper 95% CI = 2.0; predicted: pseudomedian = 2.0, lower 95% CI = 1.5, upper 95% CI = 2.0) and non-drought episode lengths (observed: pseudomedian = 3.0, lower interval = 2.5, upper 95% CI = 4.0; predicted: pseudomedian = 3.0, lower 95% CI = 2.5, upper 95% CI = 3.5) were well replicated.

**Table 1:** R-squared values (and respective standard deviations) between observed and modelled abundance for all populations of sedentary grazer species.

Species	Addo	Karoo	Kruger	Lewa	Malilangwe	Mountain Zebra	NarokDistrict	Serengeti-Mara	Umfolozi
buffalo	0.70 (0.29)	–	–	0.38 (0.16)	–	0.49 (0.30)	0.20 (0.22)	0.70 (0.15)	–
hartebeest	0.49 (0.27)	0.56 (0.29)	–	0.16 (0.17)	0.46 (0.25)	0.25 (0.17)	0.40 (0.23)	–	–
impala	–	–	–	0.31 (0.23)	0.05 (0.08)	–	0.37 (0.30)	–	0.37 (0.26)
waterbuck	–	–	0.02 (0.02)	0.03 (0.02)	0.37 (0.12)	–	0.34 (0.16)	–	0.19 (0.05)

Population projections for the four species in group SG showed that average population growth rates (*λ*) decreased, while extinction probabilities (*E*) and the variation in average growth rates steadily increased, when successively considering scenario 20C, B1 and A2 (Table 2; Table S4). There was however little variation in projected average growth rates and extinction risks between scenarios B1 and A2, likely due to the late onset of increases in drought intensity. Waterbuck populations had extremely low negative average growth rates (*λ*) even under continued current conditions (scenario 20C), with the average chance of going extinct at the end of this century being 100%. Contrastingly, buffalo and impala displayed positive projected average growth rates, with both species showing a negligible drought-related average risk of extinction under all scenarios (Table 2). Hartebeest populations showed negative projected average growth rates under all scenarios, with a relatively high risk of extinction (51.1%) under continued current conditions which increased to 66.4% and 69.1% under scenarios B1 and A2 respectively (Table 2). In addition, our results also showed that smaller populations of all species will be put at higher risk of extinction from increasing future drought occurrence than larger populations (Table S4 and Table S5).

**Table 2:** Modelled average growth rate (*λ*) and mean extinction probability (*E;* in %) to 2099 across all populations of each sedentary grazer species under model scenarios 20C, B1 and A2. SD stands for standard deviation.

	Buffalo		Hartebeest		Impala		Waterbuck	
scenario	*λ* (SD)	*E*	*λ* (SD)	*E*	*λ* (SD)	*E*	*λ* (SD)	*E*
**20C**	1.05 (0.08)	0.00	0.98 (0.08)	51.1	1.00 (0.08)	0.0004	0.92 (0.07)	1.00
**B1**	1.04 (0.10)	0.0001	0.97 (0.09)	66.1	0.99 (0.09)	0.01	0.92 (0.08)	1.00
**A2**	1.04 (0.10)	0.00	0.97 (0.09)	69.1	0.99 (0.09)	0.01	0.92 (0.07)	1.00

## Discussion

Investigations into the potential future impacts of climate change on biodiversity mostly consider directional changes in environmental conditions (see e.g. [43], [44]), and studies into the effects of climate extremes are few at present. Here we provide research to help close this gap, and present a model framework to quantitatively assess the impact of potential future increases in such highly variable and devastating events. Our study shows that future climate change will negatively impact certain ungulate species in arid and semi-arid environments, dramatically increasing extinction risk from drought occurrence for some of them. Our results also provide support to the hypotheses that the species most at risk from increasing future drought intensity are those that are relatively sedentary, and that are wholly or partially dependent on drought-intolerant food species (e.g., grazers and mixed feeders).

Our findings that sedentary ungulate species (as opposed to nomadic or migratory species), which are dependent on drought-intolerant food species (as opposed to browsers or omnivorous species) are more at risk from current and future drought conditions are in line with frequent reports in the literature of species exhibiting these life history traits suffering high mortality during individual drought events [13], [20]–[22]. Our results suggest that at present the frequency and intensity of drought occurrence is sufficiently low that it is not inflicting a significantly negative impact on populations that are currently able to escape the effects of resource depletion in dry conditions. However, as drought intensity increases under future climate change its impact on such species may become significant.

The results of the predictive stochastic population models show that hartebeest and waterbuck will be put at extremely high risk from future increases in drought intensity under climate change over this century. Conversely, the chance of buffalo and impala populations going extinct will remain low. Those patterns are likely to reflect reported general population trends for these species throughout their global ranges, as well as trends occurring at the level of the study populations. In fact, population trends for hartebeest, buffalo and waterbuck across their global ranges are reported by the IUCN to be generally decreasing at present [45]–[47]. In particular, according to our simulations, waterbuck have an extremely high probability of extinction and low average population growth rates even under continued present conditions. While reported decline in the species is often largely due to poaching [46], the species is also one of the most water-dependent of African ungulate species and has a high-protein dietary requirement [48]. As such it is extremely susceptible to drought conditions, explaining our simulations’ results. The positive average growth rates and low risk of extinction displayed by buffalo under all scenarios, however, does not necessarily show an immunity of this species to drought conditions. Instead, the impact of drought on buffalo population dynamics could be dampened, or even counteracted, by alternative processes. For example, all study populations occur in large national parks where populations are doing well, with an increasing population trend in Addo National Park, South Africa, Mountain Zebra National Park, South Africa and Lewa Nature Conservancy, Kenya since 1991, 2002 and 1990 respectively [49], [50]. In addition, the only years in which abundance data are available for two consecutive years for the population in the Serengeti-Mara Ecosystem, Tanzania occur over 1970–1977 when the population was undergoing a rapid comeback following the eradication of the rinderpest disease in the region [51]. As a result, our population projections for buffalo under the three scenarios over the 21^st^ century may be considerably modest.

The results presented here do have some limitations. First, sample size was small for some of our study species due to populations being omitted for reasons listed above. Second, in the absence of data regarding potential alternative processes that may be acting on the study populations, the main assumption of this work is that drought intensity is the sole process acting on growth rates. However, management practices could also be acting on the population dynamics of studied populations, as many of them occur within protected areas and national parks and management actions are often listed as ‘unknown’ within the LPI database. For example, water- or food-provisioning [52]–[54], culling [21] or fencing [55]–[57] may serve to either increase or decrease the resilience of ungulate populations to drought occurrence. Similarly, processes such as poaching (particularly in the case of the black rhinoceros; [58]), predation [54], [59], dispersal [13], [60], disease outbreaks [61], [62], or the impacts of other extreme natural events (e.g., wild fire or flooding; [5]), which also have an effect on some ungulate populations, cannot be accounted for in our data. The influence of such processes could be buffering the impact of the effect of drought on populations of our study species. Third, estimation methods of yearly abundance within our dataset also differed between individual populations of our study species, with some methods resulting in coarse resolution data (e.g., scaling-up from walking transects), which may similarly have buffered the effect of drought occurrence on these populations. Individual population size estimates may also suffer some degree of inaccuracy due to the difficult nature of obtaining counts for game species [63]–[66]. Finally, the population models built to project species’ abundance under future climate change scenarios remain relatively simple. For example, they ignore processes such as density-dependence, predation or the difference in survival and reproduction rates of individuals of different sexes and ages, which are known to impact population dynamics [67]–[69]. While our results provide a good first model of variation in future population abundance in response to drought, we acknowledge that additional more complex studies will be required in order to gain a complete understanding of the potential future impact of increases in drought intensity under climate change on the persistence of ungulate populations.

In the face of our results, should highly sedentary, grazing and mixed feeding ungulate species be targeted by park authorities in the future for management in drought years? Some such species are clearly highly susceptible, but potential management strategies for their benefit may in reality come at a cost to them and to other ungulate species in arid and semi-arid environments. Studies have found that provisioning with artificial water-holes can enable less sedentary species to expand their ranges within national parks and can promote increases in these populations, in turn resulting in heightened ungulate densities at such water points, and negatively impacting rarer sedentary species such as the waterbuck, roan antelope (*Hippotragus equinus*) and tsessebe (*Damaliscus lunatus*) through resource exhaustion and increased predation [53], [54], [70]. This effect has also been found to extend to the predators of ungulate species, with lions (*Panthera leo*) in the Kruger National Park, South Africa, benefitting from higher prey densities around artificial water-points and causing competitive exclusion of the less common brown hyena (*Hyaena brunnea*) [71]. Indeed, the cause for caution in determining effective spacing of artificial water-points for the maintenance of ungulate abundance and diversity, and ecosystem heterogeneity both within and outside protected areas has been highlighted extensively in the literature [53], [72], [73]. Such provisioning can also disrupt movement patterns of migratory ungulate species, and result in heightened inter-specific competition and die-off populations of these in dry years [20]. This raises the issue of whether the risk of increasing drought severity and frequency over the 21^st^ century [9] may potentially further exacerbate the effect of artificial water-holes on both the competitive exclusion of rarer, more sedentary ungulate species and the dry season survival of migratory species. Climate change is predicted to alter the timing of migrations and the migration routes of terrestrial mammals, through altering the distribution of forage and surface-water availability [74]–[76]. Hence under future increases in drought intensity and frequency, both wet and dry season ranges of migratory ungulate species may become less able to support these populations and may be altered, which could have serious implications for the future conservation of such species. Fencing around national parks can have a severe impact on the survival of the migratory populations within, disrupting migratory pathways and heightening the impact of drought through disabling such populations to access their dry season ranges [55]–[57]. Under increasing future drought intensity, national parks should focus on the effective spacing of artificial water-points and on enabling greater connectivity for migratory ungulates. Such management strategies would assist in limiting the negative impacts of water-provisioning on both sedentary and migratory populations.

Altogether, our work illustrates that climate change and increased drought conditions could lead to the extinction of certain populations over the 21^st^ century. Our findings provide further evidence that increasing future drought conditions will pose a greater risk to ungulate species that are highly sedentary, and that are wholly or partially dependent on drought-intolerant food species. Although none of our study species are threatened species and have been classified by the International Union for the Conservation of Nature (IUCN) as ‘Least Concern’ [45]–[47], [77], our findings may have implications for some other highly threatened ungulate species and subspecies in areas where drought intensity is predicted to increase over the 21^st^ century, such as Mountain zebra (*Equus zebra*), European bison (*Bison bonasus*), Cuvier’s gazelle (*Gazella cuvieri*) and Tora hartebeest (*Alcelaphus buselaphus tora*) [78]–[81]. In addition, these drought-intolerant life history traits are also likely to cause enhanced susceptibility to increasing future drought intensity of certain species in other taxonomic groups. Our study clearly stresses the importance of long-term monitoring in order to provide a basis on which to explore the impacts of extreme natural events on animal populations under future climate change. Future studies should be conducted in order to determine the susceptibility of species in differing environments and taxonomic groups, particularly threatened species and small or isolated populations, to these increased climate extremes, in order to develop appropriate management strategies.

## Supporting Information

Figure S1 ***C*** **as a predictor of growth rates** ***r*** **for all (a) sedentary, browsing species and (b) migratory or nomadic, grazing or mixed-feeding species.** *C is* the maximum number of consecutive months of the preceding year in which *q*<*θ.* For sedentary browsing species, there were six species, n = 373, slope = −0.01, *p* = 0.20. For migratory or nomadic, grazing or mixed feeding species, there were 2 species, n = 165, slope = 0.001, *p* = 0.88.
(DOCX)

Table S1 **Ungulate species and populations used in this study (WWF/ZSL 2012).**
(DOC)

Table S2 **Probabilities under current conditions of a given year being in drought, and of different lengths of drought occurring if that’s the case, for sedentary grazing or mixed feeding species.** (a) Observed probabilities under current conditions of a given year being in drought (initial threshold; *C* >0) and not in drought (*C = *0) and that if one year is a drought year that the following year will also be a drought year (the drought distance threshold; ddt) or if one year is a non-drought year that the following year will also be a non-drought year (the non-drought distance threshold; nddt), across all populations of all sedentary grazing or mixed feeding species. (b) Observed probabilities under current conditions that if a given year is a drought year, *C* will be equal to each value between 1 and 12, across all populations of all sedentary grazing or mixed feeding species.
(DOC)

Table S3 **Spearman’s rank correlation analysis between the four predictor variables of drought intensity (** ***C, T, C_t_***_**2**_ **and** ***T_t_***_**2**_ **all** ***p*** **<0.001).**
(DOC)

Table S4 **Modelled average growth rate (** ***λ*** **) and extinction probability (** ***E*** **) of all populations of sedentary grazer species to 2099 under scenarios 20C, B1 and A2.** SD stands for standard deviation.
(DOC)

Table S5 **Relative starting abundance of populations of sedentary, grazing species (SG; relative abundance here is initial population abundance at year** ***t*** **in comparison to the initial abundance at year** ***t*** **for all populations of that species).** Due to confidential data sources, raw abundance values cannot be published.
(DOC)
