# Supplementary material for: Predicting the Future Impact of Droughts on Ungulate Populations in Arid and Semi-Arid Environments
Source: PLoS One. 2012 Dec 17;7(12):e51490. doi: 10.1371/journal.pone.0051490 (PMC3524186; doi:10.1371/journal.pone.0051490)
Supplement: Figure S1 — C as a predictor of growth rates r for all (a) sedentary, browsing species and (b) migratory or nomadic, grazing or mixed-feeding species. C is the maximum number of consecutive months of the preceding year in which q<θ. For sedentary browsing species, there were six species, n = 373, slope = −0.01, p = 0.20. For migratory or nomadic, grazing or mixed feeding species, there were 2 species, n = 165, slope = 0.001, p = 0.88. (DOCX) [file pone.0051490.s001.docx]

**Figure S1**.

(a)

(b)
