# Supplementary material for: Predicting the Future Impact of Droughts on Ungulate Populations in Arid and Semi-Arid Environments
Source: PLoS One. 2012 Dec 17;7(12):e51490. doi: 10.1371/journal.pone.0051490 (PMC3524186; doi:10.1371/journal.pone.0051490)
Supplement: Table S1 — Ungulate species and populations used in this study (WWF/ZSL 2012). (DOC) [file pone.0051490.s002.doc]

**Table S1.**

| **Species** | **N** | **Population** |
| --- | --- | --- |
| **African buffalo**  **(*Syncerus caffer*)** | 37 | Addo Elephant National Park, South Africa  Lewa Nature Conservancy, Kenya |
|  |  | Mountain Zebra National Park, South Africa |
|  |  | Narok District, Kenya |
|  |  | Serengeti-Mara ecosystem, Tanzania |
| **African bush elephant**  **(*Loxodonta africana*)** | 71 | Addo Elephant National Park, South Africa [2 estimates] |
|  |  | Kidepo Valley National Park, Uganda  Lake Manyara National Park, Tanzania |
|  |  | Malilangwe Conservancy, Zimbabwe  Mara Reserve, Kenya |
|  |  | Narok District, Kenya |
|  |  | Samburu and Buffalo Springs National Reserves, Kenya |
|  |  | Serengeti woodlands, Tanzania |
| **Black rhinoceros (*Diceros bicornis*)** | 94 | Addo Elephant National Park, South Africa  Akagera National Park, Rwanda  Hluhluwe Umfolozi Provincial Nature Reserve, South Africa  Karoo National Park, South Africa  Lake Manyara National Park, Tanzania  Lake Nakuru National Park, Kenya  Lewa Nature Conservancy, Kenya  Malilangwe Conservancy, Zimbabwe  Nairobi National Park, Kenya  Ngorongoro crater, Tanzania  Nguila Rhino Sanctuary, Tsavo West National Park, Kenya  Pilanesburg National Park, South Africa  South Luangwa National Park, Zambia |
| **Blue wildebeest**  **(*Connochaetes taurinus*)** | 116 | Hluhluwe Umfolozi Provincial Nature Reserve, South Africa  Malilangwe Conservancy, Zimbabwe  Ngorongoro Crater, Tanzania [dry season and wet season estimates]  Pilanesburg Nature Reserve, South Africa  Sabi-Sand Wiltuin, Kruger National Park, South Africa  Serengeti-Mara ecosystem, Tanzania [2 estimates]  Serengeti plains, Tanzania |
| **Common warthog (*Phacochoerus africanus*)** | 57 | Addo Elephant National Park, South Africa  Kasanka National Park, Zambia  Kruger National Park, South Africa  Lewa Nature Conservancy, Kenya  Malilangwe Conservancy, Zimbabwe |
| **Giraffe**  **(*Giraffa camelopardalis* )** | 54 | Burigi Game Reserve, Tanzania  Hluhluwe Umfolozi Provincial Nature Reserve, South Africa  Kruger National Park, South Africa  Lake Manyara National Park, Tanzania  Lake Nakuru National Park, Kenya  Lewa Nature Conservancy, Kenya  Malilangwe Conservancy, Zimbabwe  Narok District, Kenya |
| **Greater kudu (*Tragelaphus strepsiceros*)** | 48 | Addo Elephant National Park, South Africa  Karoo National Park, South Africa  Kruger National Park, South Africa  Malilangwe Conservancy, Zimbabwe  Mountain Zebra National Park, South Africa  Hluhluwe Umfolozi Provincial Nature Reserve, South Africa |
| **Hartebeest**  **(*Alcelaphus buselaphus*)** | 40 | Addo Elephant National Park, South Africa  Karoo National Park, South Africa  Lewa Nature Conservancy, Kenya  Malilangwe Conservancy, Zimbabwe  Mountain Zebra National Park, South Africa  Narok District, Kenya |
| **Iberian ibex (*Capra pyrenaica*)** | 46 | Amarela-Santa Eufemia mountain range, Portugal  Central part of the Geres-Xures mountain range, Portugal  Eastern limit of the Geres-Xures mountain range, Portugal  Gredos, Spain  Sierras de Cazorla y Segura, southern Spain  Tejada-Almijara, Andalucia, Spain  Tortosa-Beceite, Spain |
| **Impala**  **(*Aepyceros melampus*)** | 26 | Hluhluwe Umfolozi Provincial Nature Reserve, South Africa  Lewa Nature Conservancy, Kenya  Malilangwe Conservancy, Zimbabwe |
|  |  | Narok District, Kenya |
| **Waterbuck**  **(*Kobus ellipsiprymnus*)** | 42 | Hluhluwe Umfolozi Provincial Nature Reserve, South Africa  Kruger National Park, South Africa |
|  |  | Lewa Nature Conservancy, Kenya |
|  |  | Malilangwe Conservancy, Zimbabwe |
|  |  | Narok District, Kenya |
| **Wild boar**  **(*Sus scrofa*)** | 36 | Castelporziano Reserve, Italy  Lovat River head, Gorodok district, Vitebsk region, northeastern Belarus  Monticiano, Seina, Italy  Western Poland |
| **N = sample size of growth rate between two consecutive years ($r$) for each species** | | |
