# Supplementary material for: Predicting the Future Impact of Droughts on Ungulate Populations in Arid and Semi-Arid Environments
Source: PLoS One. 2012 Dec 17;7(12):e51490. doi: 10.1371/journal.pone.0051490 (PMC3524186; doi:10.1371/journal.pone.0051490)
Supplement: Table S2 — Probabilities under current conditions of a given year being in drought, and of different lengths of drought occurring if that’s the case, for sedentary grazing or mixed feeding species. (a) Observed probabilities under current conditions of a given year being in drought (initial threshold; C >0) and not in drought (C = 0) and that if one year is a drought year that the following year will also be a drought year (the drought distance threshold; ddt) or if one year is a non-drought year that the following year will also be a non-drought year (the non-drought distance threshold; nddt), across all populations of all sedentary grazing or mixed feeding species. (b) Observed probabilities under current conditions that if a given year is a drought year, C will be equal to each value between 1 and 12, across all populations of all sedentary grazing or mixed feeding species. (DOC) [file pone.0051490.s003.doc]

**Table S2**.

(a)

| threshold | probability |
| --- | --- |
| **initial threshold (*C* > 0)**  ***C* = 0** | 0.30  0.70 |
| **ddt** | 0.45 |
| **ddt** | 0.74 |

(b)

| *C* | probability |
| --- | --- |
| **1** | 0.24 |
| **2** | 0.27 |
| **3** | 0.15 |
| **4** | 0.10 |
| **5** | 0.05 |
| **6** | 0.04 |
| **7** | 0.05 |
| **8** | 0.02 |
| **9** | 0.02 |
| **10** | 0.02 |
| **11** | 0.03 |
| **12** | 0.01 |
