# Supplementary material for: Predicting the Future Impact of Droughts on Ungulate Populations in Arid and Semi-Arid Environments
Source: PLoS One. 2012 Dec 17;7(12):e51490. doi: 10.1371/journal.pone.0051490 (PMC3524186; doi:10.1371/journal.pone.0051490)
Supplement: Table S3 — Spearman’s rank correlation analysis between the four predictor variables of drought intensity ( C, T, Ct 2 and Tt 2 all p <0.001). (DOC) [file pone.0051490.s004.doc]

**Table S3**.

|  | $C$ | $T$ | $C_{t2}$ | $T_{t2}$ |
| --- | --- | --- | --- | --- |
| $C$ | 1.000 | 0.998 | 0.737 | 0.746 |
| $T$ | 0.998 | 1.000 | 0.736 | 0.749 |
| $C_{t2}$ | 0.737 | 0.736 | 1.000 | 0.992 |
| $T_{t2}$ | 0.746 | 0.749 | 0.992 | 1.000 |
