# Supplementary material for: Predicting the Future Impact of Droughts on Ungulate Populations in Arid and Semi-Arid Environments
Source: PLoS One. 2012 Dec 17;7(12):e51490. doi: 10.1371/journal.pone.0051490 (PMC3524186; doi:10.1371/journal.pone.0051490)
Supplement: Table S4 — Modelled average growth rate ( λ ) and extinction probability ( E ) of all populations of sedentary grazer species to 2099 under scenarios 20C, B1 and A2. SD stands for standard deviation. (DOC) [file pone.0051490.s005.doc]

**Table S4**.

| population |  | **Buffalo** | | **Hartebeest** | | **Impala** | | **Waterbuck** | |
| --- | --- | --- | --- | --- | --- | --- | --- | --- | --- |
| scenario | | $\lambda$ (SD) | $E$ | $\lambda$ (SD) | $E$ | $\lambda$ (SD) | $E$ | $\lambda$ (SD) | $E$ |
| Addo | **20C** | 1.05 (0.08) | 0.00 | 0.98 (0.07) | 0.96 | - | - | - | - |
|  | **B1** | 1.04 (0.10) | 0.00 | 0.97 (0.09) | 0.997 | - | - | - | - |
|  | **A2** | 1.04 (0.10) | 0.00 | 0.96 (0.09) | 0.998 | - | - | - | - |
| Karoo | **20C** | - | - | 0.98 (0.08) | 0.03 | - | - | - | - |
|  | **B1** | - | - | 0.96 (0.09) | 0.32 | - | - | - | - |
|  | **A2** | - | - | 0.96 (0.09) | 0.41 | - | - | - | - |
| Kruger | **20C** | - | - | - | - | - | - | 0.92 (0.07) | 1.00 |
|  | **B1** | - | - | - | - | - | - | 0.91 (0.08) | 1.00 |
|  | **A2** | - | - | - | - | - | - | 0.92 (0.08) | 1.00 |
| Lewa | **20C** | 1.05 (0.08) | 0.00 | 0.98 (0.08) | 0.97 | 1.00 (0.08) | 0.002 | 0.92 (0.07) | 1.00 |
|  | **B1** | 1.04 (0.10) | 0.0002 | 0.97 (0.09) | 0.996 | 0.99 (0.09) | 0.04 | 0.92 (0.07) | 1.00 |
|  | **A2** | 1.04 (0.10) | 0.00 | 0.97 (0.09) | 0.998 | 0.99 (0.09) | 0.05 | 0.92 (0.07) | 1.00 |
| Malilangwe | **20C** | - | - | 0.98 (0.07) | 0.998 | 1.00 (0.08) | 0.00 | 0.92 (0.07) | 1.00 |
|  | **B1** | - | - | 0.97 (0.09) | 1.00 | 0.99 (0.09) | 0.00 | 0.92 (0.08) | 1.00 |
|  | **A2** | - | - | 0.97 (0.09) | 1.00 | 0.99 (0.10) | 0.0002 | 0.92 (0.07) | 1.00 |
| Mountain Zebra | **20C** | 1.05 (0.08) | 0.00 | 0.98 (0.08) | 0.12 | - | - | - | - |
|  | **B1** | 1.04 (0.10) | 0.0004 | 0.96 (0.09) | 0.58 | - | - | - | - |
|  | **A2** | 1.04 (0.10) | 0.00 | 0.96 (0.09) | 0.64 | - | - | - | - |
| Narok District | **20C** | 1.05 (0.08) | 0.00 | 0.98 (0.08) | 0.001 | 1.00 (0.08) | 0.00 | 0.92 (0.07) | 1.00 |
|  | **B1** | 1.04 (0.10) | 0.00 | 0.97 (0.09) | 0.07 | 0.99 (0.09) | 0.00 | 0.91 (0.08) | 1.00 |
|  | **A2** | 1.04 (0.10) | 0.00 | 0.97 (0.09) | 0.10 | 0.99 (0.09) | 0.00 | 0.92 (0.08) | 1.00 |
| Serengeti-Mara | **20C** | 1.05 (0.08) | 0.00 | - | - | - | - | - | - |
|  | **B1** | 1.04 (0.10) | 0.00 | - | - | - | - | - | - |
|  | **A2** | 1.04 (0.10) | 0.00 | - | - | - | - | - | - |
| Umfolozi | **20C** | - | - | - | - | 1.00 (0.08) | 0.00 | 0.92 (0.07) | 1.00 |
|  | **B1** | - | - | - | - | 0.99 (0.09) | 0.00 | 0.92 (0.08) | 1.00 |
|  | **A2** | - | - | - | - | 0.99 (0.09) | 0.0002 | 0.92 (0.07) | 1.00 |
|  | | | | | | | | |  |
