# Supplementary material for: Predicting the Future Impact of Droughts on Ungulate Populations in Arid and Semi-Arid Environments
Source: PLoS One. 2012 Dec 17;7(12):e51490. doi: 10.1371/journal.pone.0051490 (PMC3524186; doi:10.1371/journal.pone.0051490)
Supplement: Table S5 — Relative starting abundance of populations of sedentary, grazing species (SG; relative abundance here is initial population abundance at year t in comparison to the initial abundance at year t for all populations of that species). Due to confidential data sources, raw abundance values cannot be published. (DOC) [file pone.0051490.s006.doc]

**Table S5**.

|  | **Addo** | **Karoo** | **Kruger** | **Lewa** | **Malilangwe** | **Mountain Zebra** | **Narok District** | **Serengeti-Mara** | **Umfolozi** |
| --- | --- | --- | --- | --- | --- | --- | --- | --- | --- |
| buffalo | 0.001 | - | - | 0.001 | - | 0.001 | 0.22 | 0.77 | - |
| hartebeest | 0.004 | 0.09 | - | 0.005 | 0.002 | 0.04 | 0.85 | - | - |
| impala | - | - | - | 0.003 | 0.04 | - | 0.84 | - | 0.11 |
| waterbuck | - | - | 0.59 | 0.01 | 0.02 | - | 0.30 | - | 0.07 |
